# Using integrated GIS and hydrological analysis for sizing culverts of multiple channel crossings at the flooded section of the Daboya-Mankarigu Road (IR10) in Ghana

**DOI:** 10.1016/j.heliyon.2023.e22863

**Published:** 2023-11-25

**Authors:** Jeff Dacosta Osei, Peter Damoah-Afari, F.O.K. Anyemedu, Eric Olenya Lartey, Lily Lisa Yevugah

**Affiliations:** a Kwame Nkrumah University of Science and Technology, Department of Civil Engineering- Transportation Research and Education Centre Kumasi (TRECK), Ghana; b Kwame Nkrumah University of Science and Technology, Department of Civil Engineering, Kumasi, Ghana; c Kwame Nkrumah University of Science and Technology, Department of Geomatic Engineering, Kumasi, Ghana; d University of Energy and Natural Resources, Department of Geospatial Sciences, Dormaa Ahenkro, Ghana

**Keywords:** GIS, SWAT, Hydrological model, Box culvert, Pipe culvert, Peak flow, Modified rational method

## Abstract

A culvert is an important structure in Road construction to allow the conveyance of Channels crossing the road. Culverts are sized for a road to accommodate the volume of water crossing the road network to avoid flooding. Ghana Highways has a standard manual for culvert sizing at channel crossing. This manual serves as a guide for the proper sizing of culverts, however, lots of culverts have been found to have failed. Among the reasons for the failure of culverts could be under-sizing, urbanization, climate change, lack of maintenance, etc. The Daboya-Mankarigu Road is situated in the Savanah Region of Ghana in the North Gonja District. The section of the road from Chainage 9 + 075 to 10 + 200 has been experiencing flooding from 2020 to 2021 with a flood depth of 3.315 m in 2020 and 2.00 m in 2021. This study seeks to use integrated GIS and hydrological-based methods to propose new culverts to supplement the existing culverts to control flooding at section (9 + 075 to 10 + 200) of Daboya-Mankarigu Road (IR10). Geographic Information system (GIS) model (SWAT), Hydrological and hydraulic models were used to determine the peak flow at the catchment to Propose new culverts to supplement the existing culverts. Using a design period of 25 years for culverts, the modified rational method was used to determine the Peak flow of the catchment. A 25-year peak flow of 367.155 m^3^/s was determined and used for hydraulic analysis of the existing culverts. From the study, the existing culvert structures at the section had a hydraulic capacity of 78.732 m^3^/s which could not accommodate the remaining flow of 288.423 m^3^/s in the catchment. An observation was made that the changes in the rainfall can cause a change in rainfall intensity. An increase in built-up areas in the catchment can also increase the runoff coefficient which can result in higher peak flow in the catchment. Climate change, change in slope, and Land use in the catchment were also determined to have a huge influence on the adequacy of culverts since the peak flow is dependent on these parameters in the catchment as the years go by. A 4No. 4 m × 4 m box culvert, 3No. 3.5 m × 3.5 m box culvert, and 13 No. 1200 mm pipe culverts with 2 each at different chainages were proposed at suitable locations to supplement the existing culverts using the HDS-5 equations in AutoCAD Civil 3D. A recommendation is made to consider the installation of these new culverts at the flood section to control flooding and avoid overtopping of water on the IR10 road section (Daboya-Mankarigu) in the north Gonja District.

## 1 Introduction

**Fig. 1:**
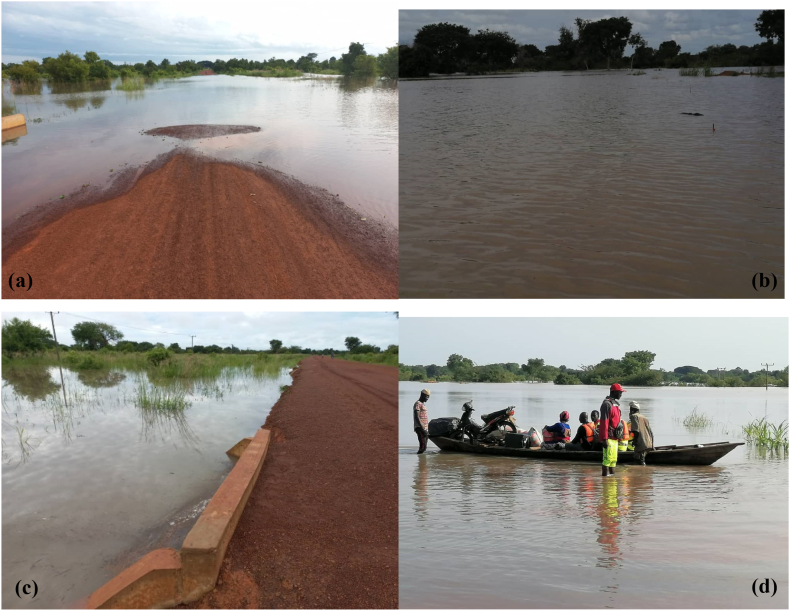
Flooded section on the IR10 road (Daboya-Mankarigu) in the North Gonja district of the savannah region in Ghana (a) Flooded road (b) Overtopped culvert (c) Insufficient culvert size at the flooded section (d) Pedestrians using a boat to cross the road.

Flooding has become a national headache for Ghana since its independence. Several cities like Accra and Kumasi have recorded several issues of flooding [1]. The Country spends a lot of money to control this challenge without paying attention to the northing part of the country. Developing road networks is a crucial priority for the Ghanaian government, but often times the maintenance culture needed to extend their lifespan is overlooked. One of the challenges faced in these road networks is the occurrence of traffic congestion caused by channel crossings, which can hamper smooth transportation. In the transportation infrastructure, culverts play a vital role as they facilitate water transportation across roadways and prevent flooding. However, their maintenance is often neglected in favour of other highway infrastructure such as pavements or bridges, resulting in some culverts becoming vulnerable to flooding and decay in Ghana. The large number of culverts along the road networks in Ghana puts a significant strain on road agencies responsible for designing, managing, and maintaining them during floods. Residents of the urban and suburbs in the country rarely consider how they would get to and from work, or any other place. Inhabitants living on a stretch of road with few homes in rural areas like the flood section of the Inter-regional road 10 (IR10) (Daboya-mankarigu road) of the country, on the other hand, are aware that if it rains, the culverts may be impassable because of flooding since 2018. A flood depth of 3.736 m as shown in Fig. 1 on the road network becomes a headache for the inhabitants every year.

When culverts are not properly sized, situations like this can occur. Designing culverts for road crossings requires careful consideration of various engineering and technical aspects both at the culvert site and the surrounding areas. In addition to technical standards, it is important for engineers to apply their own judgment and personal experience when determining the factors that should be taken into account during culvert design, as well as deciding on the appropriate dimensions for the final design [2]. A drainage culvert should be designed by using design standards to drain the design peak flow safely. Norman et al. [3] compared various culvert design methods to determine the best design method. This resource provides design methods for both conventional culverts and culverts with inlet improvements. Muste et al. [4] employed real-time data for discharge and water level data from multiple upstream and downstream locations of a culvert structure to carry out steady and unsteady flow laboratory measurements on various culvert-barrel cross-section shapes and configurations. Kang et al. [5] proposed a drainage culvert design approach that takes into account the critical storm duration. Their findings suggest that using a design flood based on the critical storm duration is more effective than using a design flood based solely on the rational method. Consequently, they advocate for the adoption of the design approach based on the concept of critical storm duration for estimating a design flood. By applying this approach to standard design in Korea, various subjective methods of estimating the design floods can be made more objective. Furthermore, they recommended that a design that accounts for anticipated changes in land use could be achieved by incorporating a Geographical Information System (GIS). Greer et al. [6] utilized a GIS-Enabled Culvert Design approach in their case study conducted in Tuscaloosa, Alabama. This approach involves the use of Python programming to design a culvert automatically by integrating the proposed culvert location, topography, land use, and rainfall data. To enhance the efficiency of this approach, the module was incorporated into the ESRI ArcGIS 10.4 software, resulting in a unified platform that eradicates the propagation of errors associated with cross-platform data transfer. Furthermore, this approach saved approximately 95 % of the time required for traditional calculation methods [5]. The module used digital elevation data from the US Geological Survey to analyze the topography of the watershed, while data from the National Land Cover Database were employed to calculate runoff coefficients. The study employed the rational method, which combines rainfall data from the National Oceanic and Atmospheric Administration with watershed and land use information to compute the peak discharge. Based on the culvert design parameters and peak discharge, a single-barrel culvert was constructed. The study's techniques, which involved using the module to redesign existing culverts based on updated land cover and rainfall conditions, could be useful for identifying vulnerabilities and planning infrastructure improvements [5]. The calculation of precise design floods for the numerous drainage culverts is a time-consuming and challenging task that is often based on rough calculations due to the high number of culverts. However, inaccurate calculations of design floods can have a significant impact on safety and construction costs. Therefore, the use of a Geographical Information System (GIS) has emerged as a powerful tool to tackle this problem. Although GIS was initially developed in the 1970s and early 1980s for displaying maps, it has become more prevalent and is now used to support a variety of geographically related information. Günal and Güven [7,8] studied most engineering problems today, such as geomorphologic parameters of a basin and forming synthetic hydrographs. GIS has also proven to be a valuable tool for forecasting. Günal and Kösen [9] used a GIS tool to forecast underground drinking water areas in the Gaziantep region. Analysis Program (HY-8) or commercial software like Bentley Culvert Master [3]. The peak flow rate, culvert slope, headwater and tailwater elevations, and culvert barrel diameter are all design parameters for cylindrical culverts [9]. Culvert design software usually calculates and modifies parameters specific to the site, like culvert slope and headwater elevations until appropriate design conditions are achieved. However, the peak flow rate, a crucial input for hydraulic infrastructure, often needs to be performed externally before being formatted and translated. Conducting handheld hydrological analysis can be time-consuming, and data transfer can result in errors due to formatting and translation. Therefore, a hydrological analysis must be conducted separately using hydrologic modelling software, GIS, or a combination of both.

The traditional approach for calculating the design flood has been the rational method, which only considers the peak runoff rate. However, a more accurate hydrograph-based method is recommended to calculate the design flood. In addition, the frequency-based rainfall method used in existing standard designs is inadequate, and a more precise method that considers rainfall duration distribution is necessary. The rational method uses the time of concentration as the maximum average rainfall intensity in most standard designs, but this may not be accurate in cases where highly impervious areas contribute to a greater peak discharge [10,11]. This suggests that the existing rational method has limitations, such as problems with time of concentration calculation, and that the concept of critical storm duration is being considered as an alternative. The critical storm duration is used to determine the maximum peak discharge, and this value is used to size the culverts [12].

There have been several uses of traditional culvert sizing techniques and software. Software programs such as the Culvert Hydraulic Analysis Program of the Federal Highway Administration (FHWA) are examples of traditional culvert design software [13] or commercial programs such as Bentley Culvert Master [14]. The use of hydrological analysis using models like WinTR-20 and WinTR-55 [15], HEC-HMS [16], and many more. Most of these hydrological analyses were used without a Geographic information system (GIS). A manual calculation is done and the parameters are fed into the model to determine the peak flow for culvert sizing [15]. Many tools and functions are available on modern GIS platforms for hydrologic analysis. The embedded coupling of a hydrologic model eliminates the need to maintain multiple programs and the issues associated with cross-platform data transfer. The use of an integrated GIS environment is an effective and time-saving tool for hydrological analysis for culvert sizing. Therefore, this study seeks to use integrated GIS and hydraulic models to propose new culverts to supplement the existing culverts to control flooding at the vulnerable section on the Daboya-Mankarigu Road (IR10) in Ghana.

## 2 Materials and methods

### 2.1 Study area

**Fig. 2:**
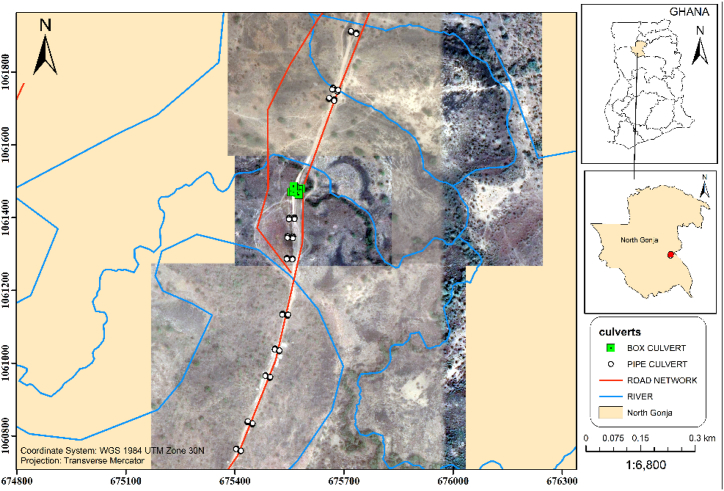
A map of the flood section on the IR10 in the north Gonja District in Ghana.

This study looks at the flood section of the Daboya-Mankarigu road network in the Gonja North district of the savannah region of Ghana. The Flooded section starts from CH 9 + 075 to CH 10 + 025 where 9 + 075–10 + 200 are always in the critical stage. The location and sizes of the existing culverts are shown in Table 1. One of Ghana's 261 Metropolitan, Municipal, and District Assemblies (MMDAs), the North Gonja District is one of the seven MMDAs that make up the Savannah Region. North Gonja District, which was split off from West Gonja and has Daboya as its capital, is one of the new districts and municipalities founded in 2012 and inaugurated concurrently on June 28, 2012, throughout their various locations. The Northern Region of Ghana's Western Region includes the North Gonja District. Located between latitude 80 32′ and 100 2′ North and longitude 1° 5′ and 2° 58′ West, it has a total land area of approximately 4845.5 km^2^ or 6.9 % of the total land area of Savannah Region. Tolon District to the east, Mamprugu Moagduri and Kumbungu Districts to the north, and Central Gonja District to the south are its neighbours. To the west are West Gonja and Wa East Districts. Fig. 2 displays the study area map.

**Table 1:** Existing culvert on the IR10 road in the North Gonja district.

Chainage	Culvert type	Cell/span	width(m)	Diameter/height(m)
9 + 075	PC	1		0.9
9 + 175	PC	1		0.9
9 + 329	PC	1		1.2
9 + 388	PC	2		1.2
9 + 439	PC	2		1.2
9 + 517	BC	3	3	3
9 + 786	PC	1		1.2
9 + 815	PC	2		1.2
9 + 981	PC	1		1.2

### 2.2 Data and materials used

This study used Land Use Land cover data obtained from Copernicus for previous and impervious classification. The study obtained an SRTM DEM (Shuttle Radar Topography Mission Digital Elevation Model) with a resolution of 30 m from the US Geological Survey (USGS) and utilized it for catchment delineation through ArcGIS 10.4 with Arc SWAT software. The software comes with a Geodatabase containing weather data and soil type information. The materials and data used for the whole process with their sources are elaborated in Table 2.

**Table 2:** Materials used for the study.

Data	Source
SRTM DEM (Shuttle Radar Topography Mission Digital Elevation Model)	https://earthexplorer.usgs.gov/
IDF Intensity duration frequency curve	Ghana Meteorological Service
Average Annual Rainfall Data	Ghana Meteorological Service
Land Use Land cover data	https://land.copernicus.eu/global/products/lc
ArcGIS 10.4	https://Esri.com
Arc SWAT 2012.10.4.21	https://swat.tamu.edu/software/arcswat/
AutoCAD 2019	https://www.autodesk.com/education/edu-software/overview?sorting=featured&filters=individual

### 2.3 Methods

To incorporate GIS and hydrological processes into culvert sizing, the study employed the drainage culvert design approach for ungauged small watersheds. The catchment of the culverts at IR10 was delineated using the Soil and Water Assessment Tool (SWAT) model developed for the GIS environment. The peak flow was computed by using the hydrological parameters derived from SWAT. The processes used by the integrated GIS and hydrological model can be categorized into three phases.

(1) Soil and water assessment tool (SWAT) analysis
(2) Computation of peak flow and
(3) Culvert sizing

These processes are conducted with the drainage standard of the Ghana Ministry of Roads and Highways.

#### 2.3.1 Ministry of roads and highways policy on culverts

The Ministry of Roads and Highways has created a manual for designing drainage systems, which has been adopted by various agencies and departments such as the Ghana Highway Authority, Department of Urban Roads, Department of Feeder Roads, and Hydrological Services Department under the Ministry of Water Resources, Works, and Housing. This manual includes guidelines for designing drainage systems, which are summarized in Table 3, Table 4, and Table 5.

**Table 3:** The design rainfall frequency of occurrence for the hydrologic analysis (Department of Urban Roads manual, 2006).

Type of Structure/Drainage system	Frequency of Occurrence (years)
Side Culvert	10
Closed System Drainage	10
Cross culvert	25
Minor/medium-span bridges	50
Major/Long span bridges	100

**Table 4:** Sections of culverts.

Circular culverts/Closed System Drains	Rectangular Culverts
Minimum diameter for all culverts: 900 mm	Minimum internal height: 1000 mm
Maximum diameter for cross culvert: 1800 mm

**Table 5:** Manning's roughness.

Material	n value
Concrete lined channel	0.013–0.015
Sand Crete block	0.015–0.020
Masonry	0.017–0.030
Earth (new)	0.018–0.030
Earth (existing)	0.022–0.060

#### 2.3.2 Rainfall frequency of occurrence

The design rainfall frequency of occurrence for the hydrologic analysis is shown in Table 3.

#### 2.3.3 SWAT analysis in GIS

The SWAT concept is a valuable tool in evaluating catchment characteristics during the design of drainage culverts. Additionally, the use of SWAT is suitable for determining the Time of Concentration, flow length, catchment area, and catchment slope, which all impact the maximum peak runoff rate. To employ the SWAT method, information on weather and topography, as well as land use within the catchment, is necessary.

The SRTM DEM was used to delineate the catchment area of the existing culverts. A survey was conducted on the field to determine the dimensions of existing culverts on the flood section of the IR10 road as shown in Table 1. The locations of the culverts were determined by using a Real-time Kinematic Global positioning system (RTK-GPS). The coordinates were plotted in the GIS environment using ArcGIS 10.4. The stream flows and all the flow lengths were determined through the hydrological analysis by SWAT. The Highest and lowest elevations of the channels crossing the road section were extracted to compute the slope of the channel from the upstream to the location of the culverts.

The DEM was used to generate the slope of the area using the 3D analyst tool in the ArcGIS environment. The slope was generated percentage as the unit and reclassified using ranges of 0 %–2 % (Flat), 2 %–7 % (Average), and over 7 % (steep). The Land use Land cover (LULC) data obtained from Copernicus was reclassified to determine the vegetation coverage within the catchment. The percentage of Vegetation and average slope within the catchment were used to extract the value of the Runoff coefficient.

#### 2.3.4 Computation of peak flow

To design a culvert, the initial step is to determine the maximum flow of water, known as the design peak flow rate, that the culvert must convey. There are several methods for estimating peak flow rates in urban areas, such as regression analysis, the Unit hydrograph method, Frequency analysis, and the rational method, among others, which are lumped-parameter approaches. This study used the modified rational method because of these two reasons: its widespread use and the water catchment area (695.30 km^2^) is more than 2 km^2^. The modified rational method applies the areal reduction factor to catchment areas of more than 2 km^2^ to improve its estimate. The rational method needs to know the features of the drainage basin that are up-gradient from where a hydraulic structure is being developed. While it may not be the most complex method, the rational method is often a viable option for estimating peak flow rates for planning purposes due to the ease of obtaining the necessary input variables. Eq (1) delineates the variables as follows: Q represents the flowrate in cubic meters per second, C is the dimensionless runoff coefficient utilized in the rational method, I is the rainfall intensity measured in millimeters per hour for a storm duration equal to Tc, Tc is the time of concentration, F denotes the areal reduction factor, and A is the size of the watershed area measured in square kilometres.

(1) Q=0.278FCIA

To execute this equation, the study computed the area of the catchment from SWAT, the Areal reduction factor which uses the average annual rainfall, return period and catchment area. The rainfall intensity was obtained from the IDF curve available for the catchment location. This was done with the help of the time of concentration (Tc) and the return period. The C value was obtained from the C value table developed by Chow et al., 1988 with the help of the LULC coverage and the slope within the catchment. The detailed processes used to compute these factors shown in Eq. (1) are elaborated as follows.

#### 2.3.5 Areal reduction factor (F)

Localized rainfall analysis is commonly used but may not accurately represent spatial distribution. To address this, the areal reduction factor is used to convert point rainfall distribution to spatial distribution for each catchment. In Ghana, the areal reduction equation developed by Rodier [17] for West Africa is often used due to the absence of a specific equation for Ghana [18]. Eq. (2) was used in this study to calculate the areal reduction factor.

(2) F=1−0.001(logA)*(9*logN−0.042*P+152)

Where:

F = areal reduction factor.

N = return period of rainfall (years).

P = average annual rainfall (mm).

A = catchment area (km^2^).

An average annual rainfall of 1000 mm from the Ghana Metrological Agency (GMet) for the catchment area. A return period of 25 years [18] and a catchment area of 695.30 km^2^ derived from the DEM were used for this study. Eq. (2) was used to compute the Areal reduction factor for the catchment.

#### 2.3.6 Time of concentration

To determine the design rainfall intensity for peak flow rates, it is recommended to use an intensity with a duration close to the time of concentration of the catchment being studied. In Ghana, Bransby William's formula is commonly used for determining the time of concentration according to the drainage manual. This formula can be found in Eq. (3).

(3) Tc=58.5L/(A0.1*S)0.2

where:

L = Mainstream length (km).

A = Catchment area (km^2^).

S = Mainstream slope (m/km).

Tc = Time of concentration (hrs).

The mainstream length, catchment area and mainstream slope were obtained from SWAT analysis performed in ArcGIS 10.4. The output of the analysis is a geodatabase with all the hydrological parameters suitable for computing Tc and Q.

#### 2.3.7 Determination of rainfall intensity

**Fig. 3:**
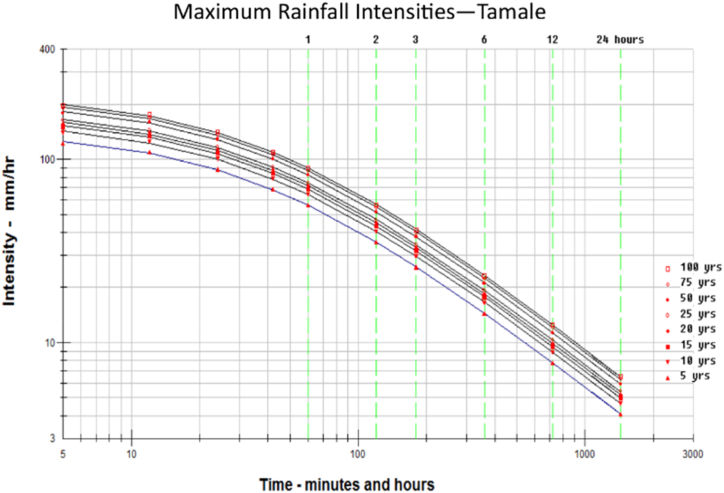
IDF curve of the catchment location (Tamale rainfall station).

Rainfall volume is a location-based meteorological characteristic that is larger in scale than a position along a flow line or even a single watershed. Climate and watershed surface factors both affect design rainfall intensity. The fundamental premise of the rational method is that peak flow happens once runoff from the most hydrologically remote point of the catchment has reached the culvert. The period when this takes place is known as the time of concentration (Tc). When assessing rainfall intensity, the rational technique frequently uses the time of concentration as a gauge of storm length. The rainfall intensity for this study was determined from the Intensity duration frequency curve (IDF curve) obtained from the Ghana metrological agency. The computed time of concentration (Tc) together with the return period was used to determine the rainfall intensity from the IDF curve in Fig. 3.

#### 2.3.8 Runoff coefficient

**Fig. 4:**
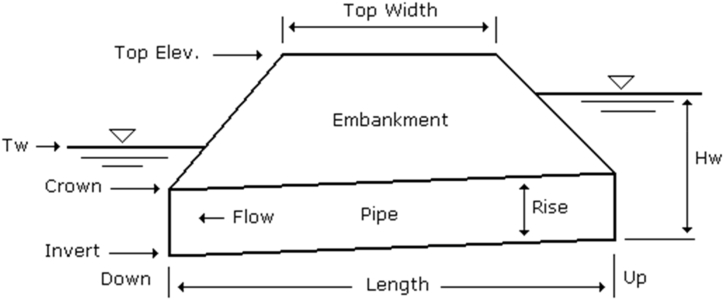
Culvert profile.

The terrain, soil type of the watershed, vegetation cover, and projected future land use patterns are used to predict the rainfall run-off coefficients. The runoff coefficient is a measure of the percentage of rainfall in a watershed that transforms into stormwater runoff. It is essential that this coefficient ranges from 0 to 1. The value of the runoff coefficient is greater in areas with low permeability. It is a dimensionless coefficient used to describe the connection between rainfall and runoff. In certain countries, the annual runoff coefficient of a basin is calculated by plotting the total annual stream flow against the total annual precipitation, and the slope of the resulting regression line is regarded as the runoff coefficient [[19], [20], [21], [22]]. The runoff coefficient, which is influenced by various factors like vegetation cover, urbanization, climate and geology, plays a crucial role in the planning, designing and operation of water resources projects in a catchment. The runoff coefficient C is determined in this study using a table provided by Chow et al. [23]. While different publications provide a range of “C" values for the rational formula, the values presented in Fig. 4 by Chow et al. [23] are considered most suitable for the grasslands and forests surrounding the Daboya-Mankarigu road in Ghana.

The runoff coefficient of this study was determined by using the vegetation cover and slope values derived from the LULC data and SRTM DEM respectively using Table 6. The slope value of 2 %–7% and Vegetation cover of more than 75 % in the catchment were used to extract runoff coefficient value using a return period of 25 years which is a requirement for the culvert from the Highway drainage design manual. The table of Runoff coefficients (Table 6) for Use in the Rational method by Chow et al. [23] was used. This table is often used in Ghana for the determination of the Runoff coefficient [18]. A runoff coefficient of 0.39 was obtained from the table using a return period of 25 years, an average slope and a grass cover larger than 75 % of the area as shown in Table 6.

**Table 6:** Runoff coefficients for Use in the Rational method [23].

	Return Period (years)		
Character of Surface	25	50	100
**Grass areas**			
Good condition (Grass cover larger than 75 % of the area)			
Flat, 0–2 %	0.29	0.32	0.36
Average, 2–7 %	0.39	0.42	0.46
Steep, over 7 %	0.44	0.47	0.51
			

#### 2.3.9 Culvert sizing using inlet control

There are two types of flow that can occur in culverts, which are known as inlet control and outlet control. Inlet control happens when the flow of water entering the pipe is restricted, causing the water to slow down as it enters. This can be compared to traffic merging from a wider road into a narrower tunnel, causing a slowdown that affects cars approaching the tunnel. Once inside the tunnel, traffic speeds up. When culverts are under inlet control, they typically have partial depth flow throughout the barrel when using Hydraflow Express Extension software. The entrance geometry of the pipe, such as its shape, area, and edge configuration, has a significant impact on inlet control. Outlet control is influenced most by factors such as n-value (barrel roughness), pipe area, shape, length, and slope, as shown in Fig. 4.

According to the Hydraulic Design Series Number 5 (HDS-5) method, the inlet control equations (**Eq. (4)** and Eq. (5)). are used to size culverts either submerged or unsubmerged. If the headwater (Hw) is above the pipe crown, the submerged equation is used [3]. This process was executed using the Hydraflow extension in Autocad Civil 3D 2019 to propose new culvert sizes for channels crossing at the flooded section of the Daboya-Mankarigu road. The adequacy of the existing culverts was analysed to determine the overtopping discharge causing the flood at the section. These new culverts are supposed to convey the overtopping discharge on the flooded section of the road.

Submerged

(4) $$\text{Hw}=D\;x\;\left[c{\left(\frac{Q}{A\sqrt{D}}\right)}^{2}+Y-0.5S\;\right]$$

Unsubmerged

(5) $$\text{Hw}=D\;x\;\left[K{\left(\frac{Q}{A\sqrt{D}}\right)}^{M}\right]$$

Where:

Hw = Headwater depth above the invert.

D = Rise or diameter, mm.

Q = Flow rate, m^3^/s.

A = Full cross-sectional area of the pipe, m^2^

K, M, c, Y = Coefficients based on edge configurations.

S = Slope, m/m.

## 3 Results and discussion

### 3.1 Catchment delineation using SWAT (hydrological analysis)

**Fig. 5:**
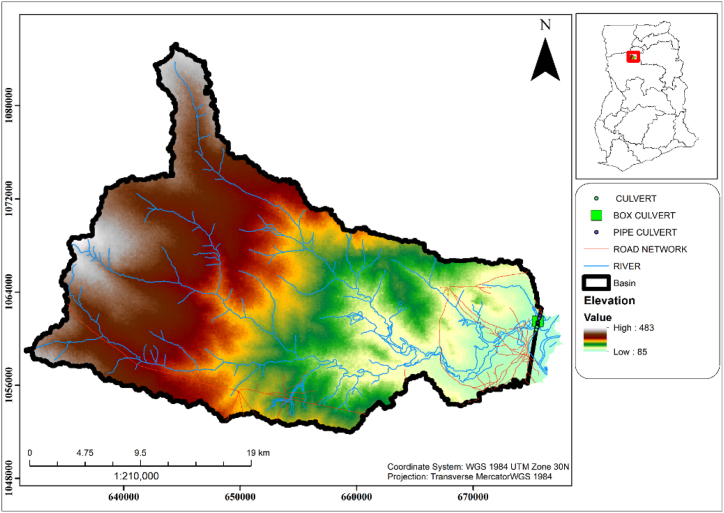
Catchment delineation of the culvert location.

**Fig. 6:**
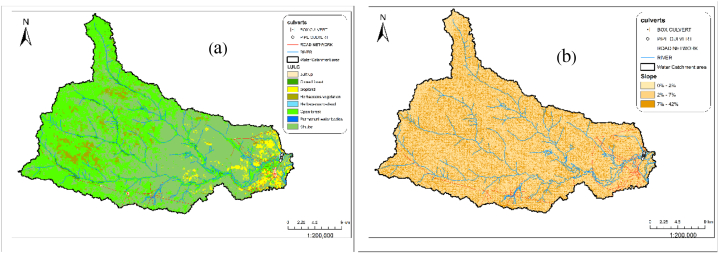
Spatial distribution of (a) Land use land cover and (b) Slope of the water catchment.

The analysis was able to produce the useful parameters shown in Table 7 required for computing the peak flow within the catchment. These parameters defined the physical characteristics of the catchment where the existing culverts can be located. It was observed that 99 % of the LULC within the catchment area is vegetation as shown in Fig. 6a. 57 % of the catchment area is within a slope of 2 %–7 % as shown in Fig. 6b. This indicates that the area is in good condition with an average slope within 2 %–7 % and vegetation cover larger than 75 % [23]. A total catchment area of 695.30 km^2^ with a flow length of 60.77 km was obtained from the SWAT analysis as shown in Table 7. It was observed that the water flows from an elevation of 483 m upstream which is the highest point to an elevation of 98 m downstream within the catchment as shown in Fig. 5.

**Table 7:** Catchment parameters extracted from SWAT analysis of the catchment area.

Catchment Area			Flow Length		H_UP_	H_down_	ΔH	Slope(S)
(m^2^)	ha	(Km^2^)	(m)	(Km)	(m)	(m)	(m)	(m/km)
695,299,702.45	695299.70	695.30	60774.21	60.77	483	98	385	6.335

### 3.2 Peak flow in the catchment

The peak flow of the catchment was obtained to be 367.155 m^3^/s for a return period of 25 years. This is a result of the time of concentration (21hrs) of the stream, the rainfall intensity, the runoff coefficient and the total area of the catchment. The rainfall intensity of 7.4 mm/h within the catchment has increased the peak flow by causing an increase in the flow depth of the stream. This flow depth has been the cause of the overtopping of the existing culverts at the section on the road. The computed parameters are shown in Table 8.

**Table 8:** Computed Peak flow at the watershed.

Tc	Tc	*i* (mm/hr)		RUN-OFF COEFF (25YRS)	RUN-OFF COEFFICIENT (50YRS)	Areal Reduction Factor	DISCHARGES	DISCHARGES
(min)	(hr)	25 years	50years	C_R25_	C_R50_	F_C_	Q (m³/s),25yrs	Q (m³/s),50ys
1277.349	21.289	7.4	8.4	0.39	0.42	0.66	367.155	448.830

### 3.3 Proposed new culvert sizes for flood control at the section

**Fig. 7:**
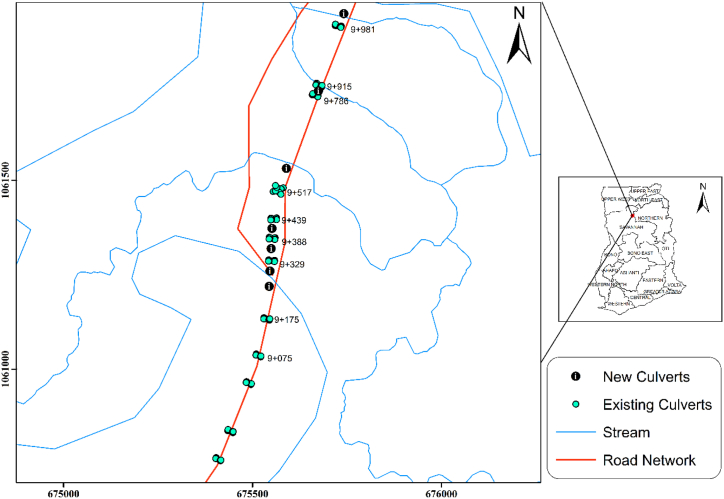
A site layout with the new and proposed culvert locations on the Flooded section.

It was observed from Table 9 that the existing culverts could only convey a flow rate of 78.732 m^3^/s instead of 367.155 m^3^/s for a 25-year return period. The remaining 288.423 m^3^/s was the overtopping flow on the road section. This clearly explained why the road has been flooding since 2019 every year. A new culvert schedule was developed for the section to aid the existing culverts. The newly proposed culverts as shown in Table 10 and Fig. 7 would improve the carrying capacity of the existing culverts by 21.369 m³/s capacity making the overall hydraulic capacity of the culverts (proposed and existing) 388.516 m³/s as shown in Table 11. This would be suitable enough to protect the road section from flooding. A culvert schedule was proposed for the flooded section as shown in Table 12.

**Table 9:** Hydraulic Analysis of Existing culverts.

Chainage	Type	Cell/Span	Width	Dia. or Height	Length	Area	Wetted perimeter	Carry capacity
			(m)	(m)	(m)	(m^2^)	(m)	(Inlet Control QD (m3/s))
8 + 862	PC	1		0.9	12	0.636	2.83	0.661
8 + 999	PC	1		0.9	12	0.636	2.83	0.661
9 + 075	PC	1		0.9	12	0.636	2.83	0.661
9 + 175	PC	1		0.9	12	0.636	2.83	0.661
9 + 329	PC	1		1.2	12	1.131	3.77	1.232
9 + 388	PC	2		1.2	12	1.131	3.77	2.464
9 + 439	PC	2		1.2	12	1.131	3.771	2.464
9 + 517	BC	3	3	3	12	9.000	12	65
9 + 786	PC	1		1.2	12	1.131	3.77	1.232
9 + 815	PC	2		1.2	12	1.131	3.77	2.464
9 + 981	PC	1		1.2	12	1.131	3.77	1.232
**Total Hydraulic Capacity (m**^**3**^**/s)**				**78.732**

**Table 10:** Hydraulics of Proposed culverts.

Chainage	Type	Cell/Span	Width	Dia. or Height	Length	A	Wp	Inlet Control QD	Total Hydraulic Capacity
			(m)	(m)	(m)	(m)	(m)	(m^3^/s)	(m^3^/s)
9 + 259	PC	2		1.2	12	1.131	3.77	2.464	
9 + 300	PC	2		1.2	12	1.131	3.77	2.464	
9 + 359	PC	2		1.2	12	1.131	2.4	2.464	
9 + 413	PC	2		1.2	12	1.131	2.4	2.464	
9 + 577	BC	4	4	4	11	16	16	200.000	
9 + 798	BC	3	3.5	3.5	12	1.131	3.77	95.000	
9 + 931	PC	2		1.2	12	1.131	3.77	2.464	
10 + 014	PC	2		1.2	12	12.25	14	2.464	**309.784**

**Table 11:** Hydraulic capacities of Proposed and existing culverts in the catchment area.

Total Hydraulic Capacity (Existing + Proposed) Q_c_	Required Hydraulic Capacity (Design) Q_R_	Net Hydraulic Capacity (Existing + Proposed - Design) Q_n_
(m³/s)	(m³/s)	(m³/s)
388.516	367.155	21.361

**Table 12:** A culvert schedule to control flooding at the IR10 (Daboya-Mankarigu) road section in North Gonja district in Ghana.

No.	Chainage	Type	No. of Barrels	Size (m)	Existing Length (m)	Proposed Length (m)	Remarks	
							Status	Instruction
1	9 + 075	PIPE	1	0.9	12	12	Existing	Maintain
2	9 + 175	PIPE	**1**	0.9	12	12	Existing	Maintain
3	9 + 259	PIPE	2	1.2	–	12	New	Install 2No. 1200 mm PC
4	9 + 300	PIPE	2	1.2	–	12	New	Install 2No. 1200 mm PC
5	9 + 329	PIPE	1	1.2	12	12	Existing	Maintain
6	9 + 359	PIPE	2	1.2	–	12	New	Install 2No. 1200 mm PC
7	9 + 388	PIPE	2	1.2	12	12	Existing	Maintain
8	9 + 413	PIPE	2	1.2	–	12	New	Install 2No. 1200 mm PC
9	9 + 439	PIPE	2	1.2	12	12	Existing	Maintain
10	9 + 517	BOX	3	3 × 3	11	11	Existing	Maintain
11	9 + 577	BOX	4	4 × 4	–	11	New	Install 4No. 4 × 4 BC
12	9 + 786	PIPE	1	1.2	12	12	Existing	Maintain
13	9 + 798	BC	3	3.5 × 3.5	–	12	New	Install 3No. 3.5 × 3.5 BC
14	9 + 815	PIPE	2	1.2	12	12	Existing	Maintain
15	9 + 931	PIPE	2	1.2	–	12	New	Install 2No. 1200 mm PC
16	9 + 981	PIPE	1	1.2	12	12	Existing	Maintain
17	10 + 014	PIPE	2	1.2	–	12	New	Install 2No. 1200 mm PC

## 4 Conclusion

Integrated Geographic Information System (GIS) and Hydraulic analysis have proven to be one of the geospatial techniques used in solving problems concerning the environment and also used in hydrological analysis. It has proven to be useful in culvert sizing with efficiency. The use of this tool could improve decision-making and construction management in the country. At the end of this study, the peak flow rate of 367.155 m^3^/s was derived from the hydrological analysis of the culvert catchment. The hydraulic capacity (78.732 m^3^/s) of the existing culvert structures at the section was also obtained from the integrated techniques. However, an increase in built-up areas within the catchment increased the runoff coefficient which resulted in higher peak flow in the catchment. Climate change and change in slope within the catchment were also determined to have a huge influence on the adequacy of culverts since the peak flow is dependent on these parameters in the catchment as the years go by. Above all, a 4No. 4 m × 4 m box culvert, 3No. 3.5 m × 3.5 m box culvert, and 13 No. 1200 mm pipe culverts with 2 each at different chainages were proposed to supplement the existing culverts. The method used in this study can be used to size culverts for channels crossing a road network. It can be used for both large and small channels. A recommendation is made to consider the installation of these new culverts at the flood section to control flooding on the Daboya-Mankarigu road in the north Gonja District.

## Funding statement

This research did not receive any specific grant from funding agencies in the public, commercial, or not-for-profit sectors.

## Data availability statement

Data included in article/supplementary material/referenced in article.

## Declaration of competing interest

The authors declare that they have no known competing financial interests or personal relationships that could have appeared to influence the work reported in this paper.
